# Antarctic fungi with antibiotic potential isolated from Fort William Point, Antarctica

**DOI:** 10.1038/s41598-022-25911-x

**Published:** 2022-12-12

**Authors:** Eunice Ordóñez-Enireb, Roberto V. Cucalón, Diana Cárdenas, Nadia Ordóñez, Santiago Coello, Paola Elizalde, Washington B. Cárdenas

**Affiliations:** 1 Laboratorio para Investigaciones Biomédicas, Facultad de Ciencias de la Vida, Escuela Superior Politécnica del Litoral, Guayaquil, Ecuador; 2 Program in Ecology, Evolution, and Conservation Biology, University of Illinois at Urbana-Champaign, Natural Resources Building 607 E. Peabody Dr., Champaign, IL 61820 USA; 3 Biochemistry and Biosupport, Research and Development, Crop Science, Bayer AG, Monheim, Germany; 4 Vaccine and Infectious Disease Organization (VIDO), University of Saskatchewan, 120 Veterinary Road, Saskatoon, SK S7N5E3 Canada; 5 School of Public Health, University of Saskatchewan, Saskatoon, SK S7N5E5 Canada

**Keywords:** Biotechnology, Drug discovery, Microbiology, Molecular biology

## Abstract

The Antarctic continent is one of the most inhospitable places on earth, where living creatures, mostly represented by microorganisms, have specific physiological characteristics that allow them to adapt to the extreme environmental conditions. These physiological adaptations can result in the production of unique secondary metabolites with potential biotechnological applications. The current study presents a genetic and antibacterial characterization of four Antarctic fungi isolated from soil samples collected in Pedro Vicente Maldonado Scientific Station, at Fort William Point, Greenwich Island, Antarctica. Based on the sequences of the internal transcribed spacer (ITS) region, the fungi were identified as *Antarctomyces* sp., *Thelebolus* sp., *Penicillium* sp., and *Cryptococcus gilvescens*. The antibacterial activity was assessed against four clinical bacterial strains: *Escherichia coli, Klebsiella pneumoniae*, *Enterococcus faecalis*, and *Staphylococcus aureus*, by a modified bacterial growth inhibition assay on agar plates. Results showed that *C. gilvescens* and *Penicillium* sp. have potential antibiotic activity against all bacterial strains. Interestingly, *Thelebolus* sp. showed potential antibiotic activity only against *E. coli*. In contrast, *Antarctomyces* sp. did not show antibiotic activity against any of the bacteria tested under our experimental conditions. This study highlights the importance of conservation of Antarctica as a source of metabolites with important biomedical applications.

## Introduction

The Antarctic continent is the coldest desert on Earth and contains nearly 90% of Earth's ice^1^. The climatic characteristics of this hostile environment include: temperatures below 0 °C, freezing and melting seasons, high UV radiation, arid conditions, and scarcity of nutrients^2–4^. Despite these harsh conditions, diverse groups of organisms have colonized the continent, with the microbiota (i.e*.* bacteria, archaea, and fungi) contributing to the most abundant biomass^5,6^. Among these, the Antarctic fungi are represented by endemic, native and cosmopolitan species, adapted to the cryosphere^7–10^.

Initial reports of Antarctic fungi were in the early twentieth century, and more than 1000 non-lichenized fungal species had been reported in this continent^11^. Nowadays, metagenomics and metabarcoding provide a pivotal contribution to biodiversity surveys in this continent and its sub-Antarctic islands^12–15^. Recent reports have described new mycological species in the maritime Antarctica region. Rosa and collaborators^12^ used metabarcoding to analyze fungal diversity in soil samples from Deception Island (South Shetland Islands). However, a significant number of sequences were only grouped at the Kingdom taxonomic level^12^. Similarly, a recent study sequenced 184 fungal taxa from the Antarctic Peninsula and South Shetland Islands, of which 37 taxa were detected for the first time in Antarctica; among maritime sampling sites, Greenwich Island showed more mycological diversity^13^.

Studies related to the diversity of fungi in Antarctica are essential to characterize Antarctic microbiology, but also to discover novel fungi metabolites. However, metabolic mechanisms for Antarctic fungi adaptation and their bioprospecting potential is still considered poorly studied^16–18^. Nevertheless, numerous researches expose the potential biotechnological applications of this kingdom, particularly in biomedicine^19–21^. This is likely due to their specialized metabolic adaptation that includes high catalytic activity at low temperatures, extracellular enzyme production, synthesis of antifreeze protein and elevated unsaturated fatty acids, among others^22,23^. Unique metabolic properties such as these result in the production of diverse secondary metabolites that are mainly regulated by internal^24,25^ and external environmental factors^26^ (*e.g.*, sexual stage, luminous intensity and pH). Thus, the extreme environment of the Antarctic continent is thought to contribute to the development of distinctive metabolites with potential antimicrobial properties that can lead to the discovery of new antibiotics^27^.

The discovery of novel compounds with bioactivity is crucial to face the increasing threat of multidrug-resistant (MDR), pandemic drug-resistant (PDR) and extensively drug-resistant bacteria (XDR)^28^, a major public health concern. To this end, microorganisms like *Streptomyces coelicolor, Amycolatopsis orientalis* and *Penicillium chrysogenum* have become valuable bioresources for the production of antibiotics^29–31^. Important medical compounds, such as beta-lactam penicillin, benzopyrenes, macrolides, and alkaloids have been isolated from fungi^32,33^. Fungi from polar regions represent a source of novel metabolites, with unique biomolecules that evolved under selective pressure^34^. Cold-adapted fungi showed antibacterial potential^35–38^, with distinctive structure and biological activity^39^. It is considered that new drugs derived from them may be currently understudied^34^, as expression of these compounds might be linked to environmental cues that are challenging to emulate under standard laboratory growth conditions^40,41^.

The present study shows the genetic, morphology and antibacterial characterization of four Antarctic fungi isolated from soil samples collected at Fort William Point, Greenwich Island, Antarctica. The phylogenetic analysis was based on sequences of the internal transcribed spacer (ITS) region and their potential antibacterial activity was assessed by a modified bacterial growth inhibition assay on agar plates.

## Results

### Fungi phylogeny

**Figure 1:**
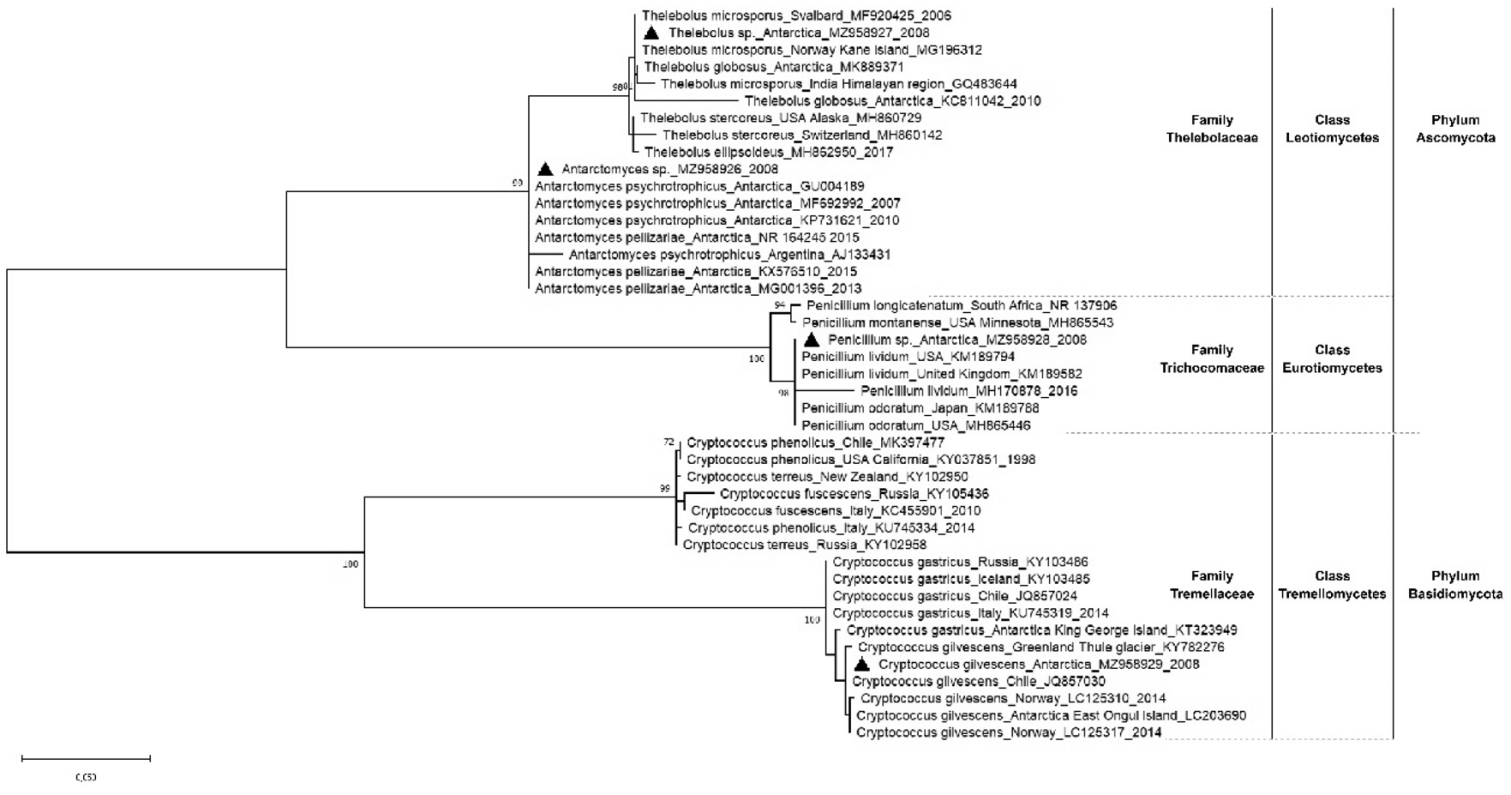
Phylogenetic tree inferred using the Maximum Likelihood estimation based on the Kimura 2-parameter model. New sequences described in this study are preceded by the symbol ▲. Taxonomic relatedness is indicated on the right side. Scale bar shows nucleotide substitutions per site. Bootstrap values higher than 70% are shown. The name of each sequence corresponds to the species, location, accession number and date, if available.

The phylogenetic analysis of the ITS sequences generated in this study (MZ958929, MZ958928, MZ958926, MZ958927) revealed that the T4-400-5E, T4-1K-1A and T4-1K-1G isolates clustered with the phylum Ascomycota, identified as *Penicillium* sp.*, Antarctomyces* sp. and *Thelebolus* sp.*,* respectively. The isolate T4-200-3B, clustered with the phylum Basidiomycete, was identified as *Cryptococcus gilvescens* (Fig. 1). All clades were strongly supported by bootstrap values higher than 70%. The tree grouped the family Thelebolaceae (*Thelebolus* and *Antarctomyces* genera) in a monophyletic group with a bootstrap value of 99% and segregated the family Trichocomaceae (*Penicillium* genus) in a separate group with a high 100% bootstrap value. *Cryptococcus gilvescens* was described as a more genetically distant species and the sequences contemplated in the tree were further divided into two additional clades with bootstraps well supported and within the family Tremellaceae.

### Morphological observations

On PDA media the macro and microscopic morphological identification of the fungi isolates corroborated the topology of the analyzed genetic sequences.

**Figure 2:**
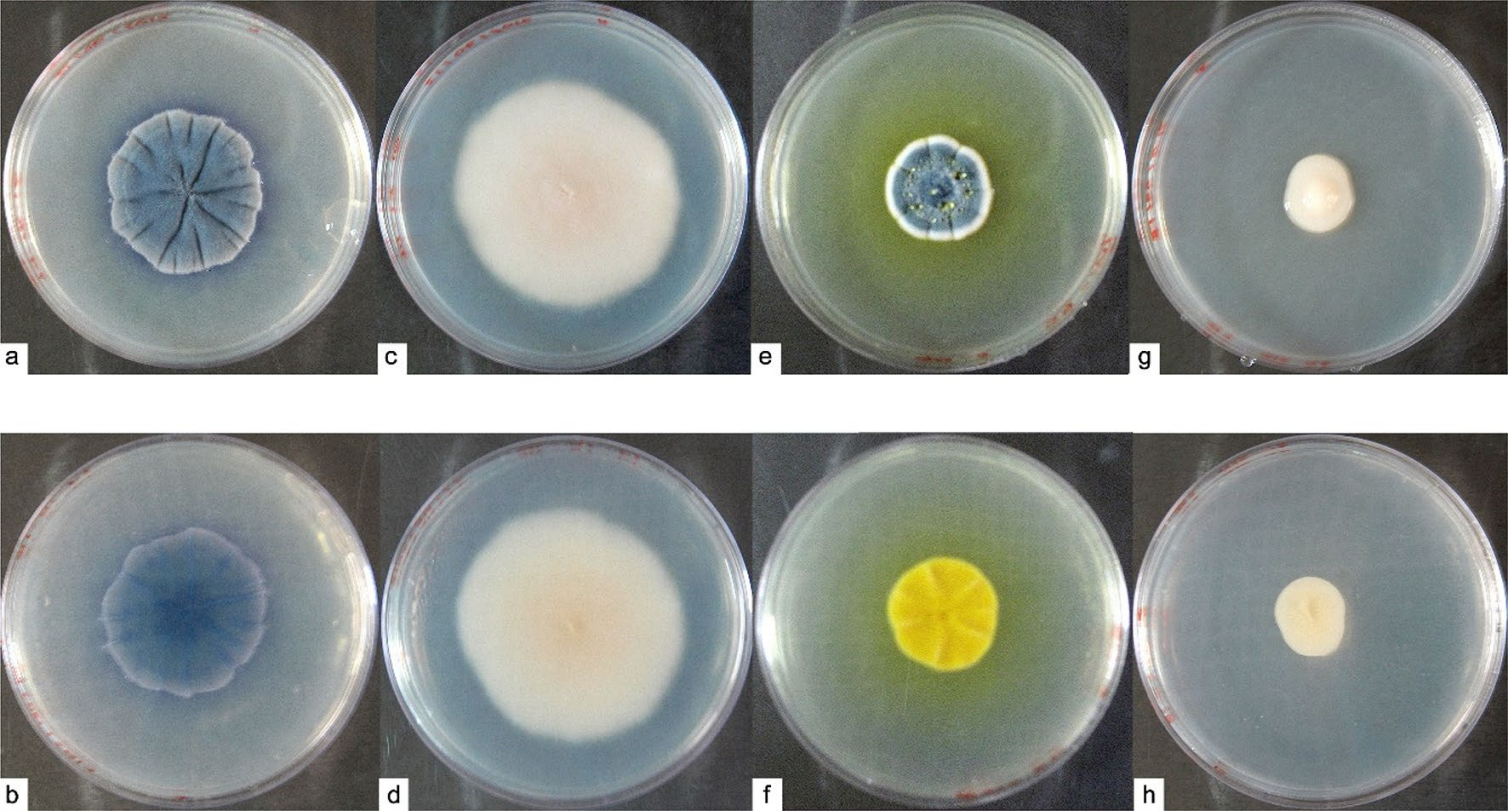
Macroscopic observation of Antarctic fungi isolates. Colonies were grown on PDA for 30 days at 4 °C. Upper panel: front of the colony. Lower panel: reverse of the colony. (**a**,**b**) *Antarctomyces* sp.; (**c**,**d**) *Thelebolus* sp.; (**e**,**f**) *Penicillium* sp.; (**g**,**h**) *Cryptococcus gilvescens*.

**Figure 3:**
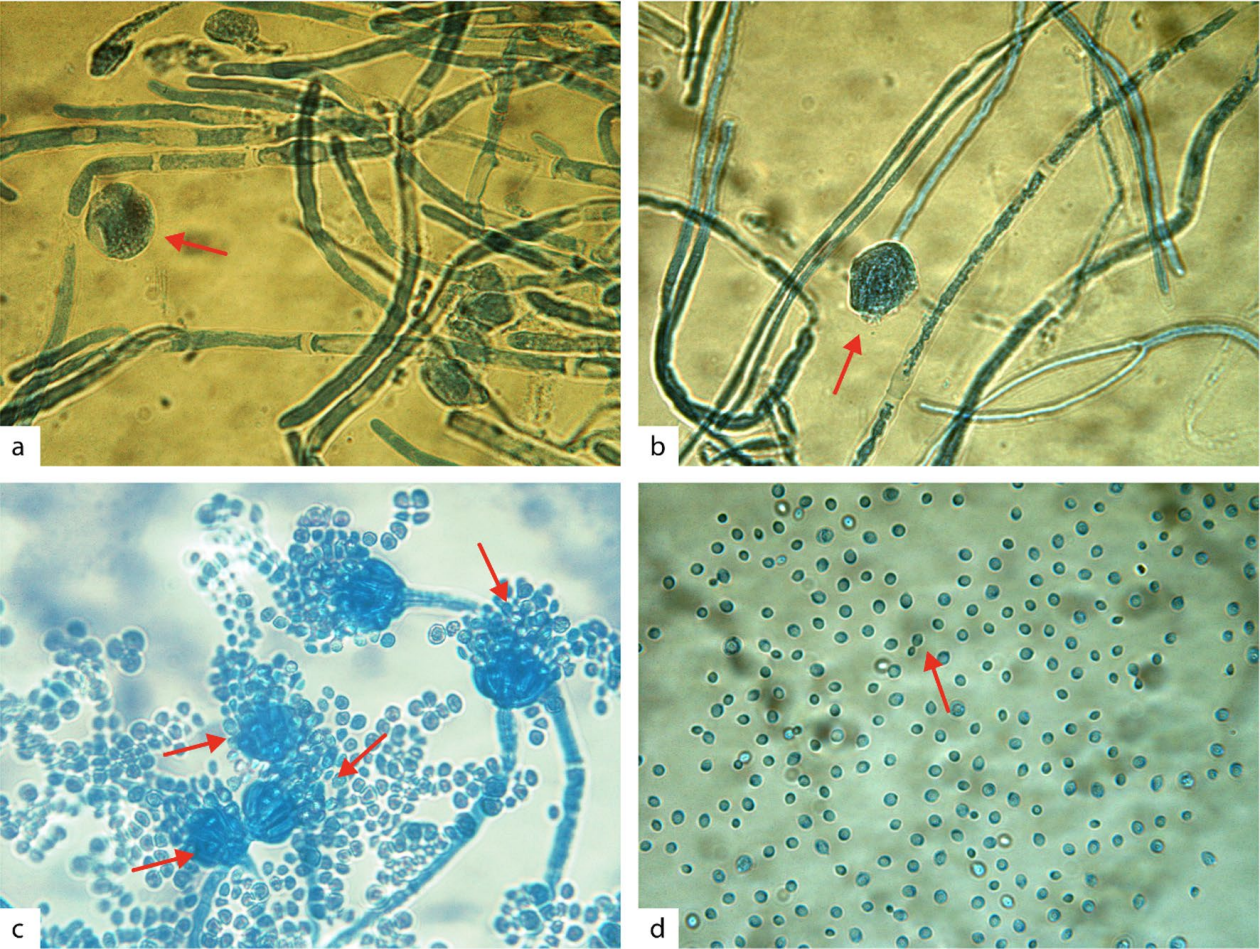
Microscopic structures of Antarctic fungi isolates observed under a compound microscope with 1000 × magnification. (**a**) *Cryptococcus gilvescens*, showing budding division of cells (arrow); (**b**) *Penicillium* sp., showing their septate stipes and conidia at the top of phialides (arrows); (**c**) *Thelebolus* sp., showing hyaline septate hyphae and ascospores (arrow); and (**d**) *Antarctomyces* sp., showing septate hyphae and ascospores (arrow). Microscopic structures were observed at 15 days (**a**), and 30 days (**b**–**d**). Scale bar 10 µm.

*Antarctomyces* sp. (T4-1K-1A) showed a smooth white colony appearance, with 3 cm of diameter after 10 days of growth. After 15 days, the colony changed to a furrowed appearance and progressively started darkening (Fig. 2a,b). At 30 days, *Antarctomyces* sp. had a blue coloration with an undulated margin and 4 cm of diameter. Microscopically, hyphae was septated with asci and immature ascospores (Fig. 3a).

*Thelebolus* sp. (T4-1K-1G) presented a circular, smooth, and cream color colony with 2 cm in size at 10 days, 3 cm and 5.5 cm of diameters after 15 and 30 days, respectively (Fig. 2c,d). Microscopically, sexual structures were scarcely developed. Immature asci and hyaline hyphae were observed (Fig. 3b).

*Penicillium* sp. (T4-400-5E) showed a yellow coloration on the agar. The colony had a furrowed appearance surrounded by a white margin. The center was umbonated with blue-green coloration. On the reverse, the colony had a yellow coloration. After 20 and 30 days of growth, the colony had a diameter of 2 cm and 2.5 cm, respectively (Fig. 2e,f). Microscopically, the anamorphic structure was represented with monoverticillate penicillin, the stipes were septated and the phialides measured 9 × 3 µm. The conidia were ovoid, with a dimension of 3.5 × 3 µm (Fig. 3c).

*Cryptococcus gilvescens* (T4-200-3B) presented a cream yeast-like colony with mucoid texture. The colony had a diameter between 1 and 1.7 cm at 15 and 30 days of growth, respectively (Fig. 2g,h). Microscopically, the cells were round to oval, with a diameter of 2.5–3 µm. Asexual reproduction by budding was observed (Fig. 3d).

### Antibiotic susceptibility tests

Standard antibiotic resistant tests with the four bacteria used in this study showed that *S. aureus* was susceptibility to all the antibiotics tested, whereas *K. pneumoniae, E. coli,* and *E. faecalis* showed resistant to three or more antibiotics (Supplementary file 1).

The modified antibacterial susceptibility test performed on *K. pneumoniae* with the PDA and temperature treatment showed that the bacteria was still resistant to imipenem (imp) and meropenem (mem) regardless of the growth conditions (Supplementary file 2). Although a slight ring of growth inhibition was noticed on PDA as compared to LB agar, it was considered still a resistant phenotype.

### Antibacterial potential

**Figure 4:**
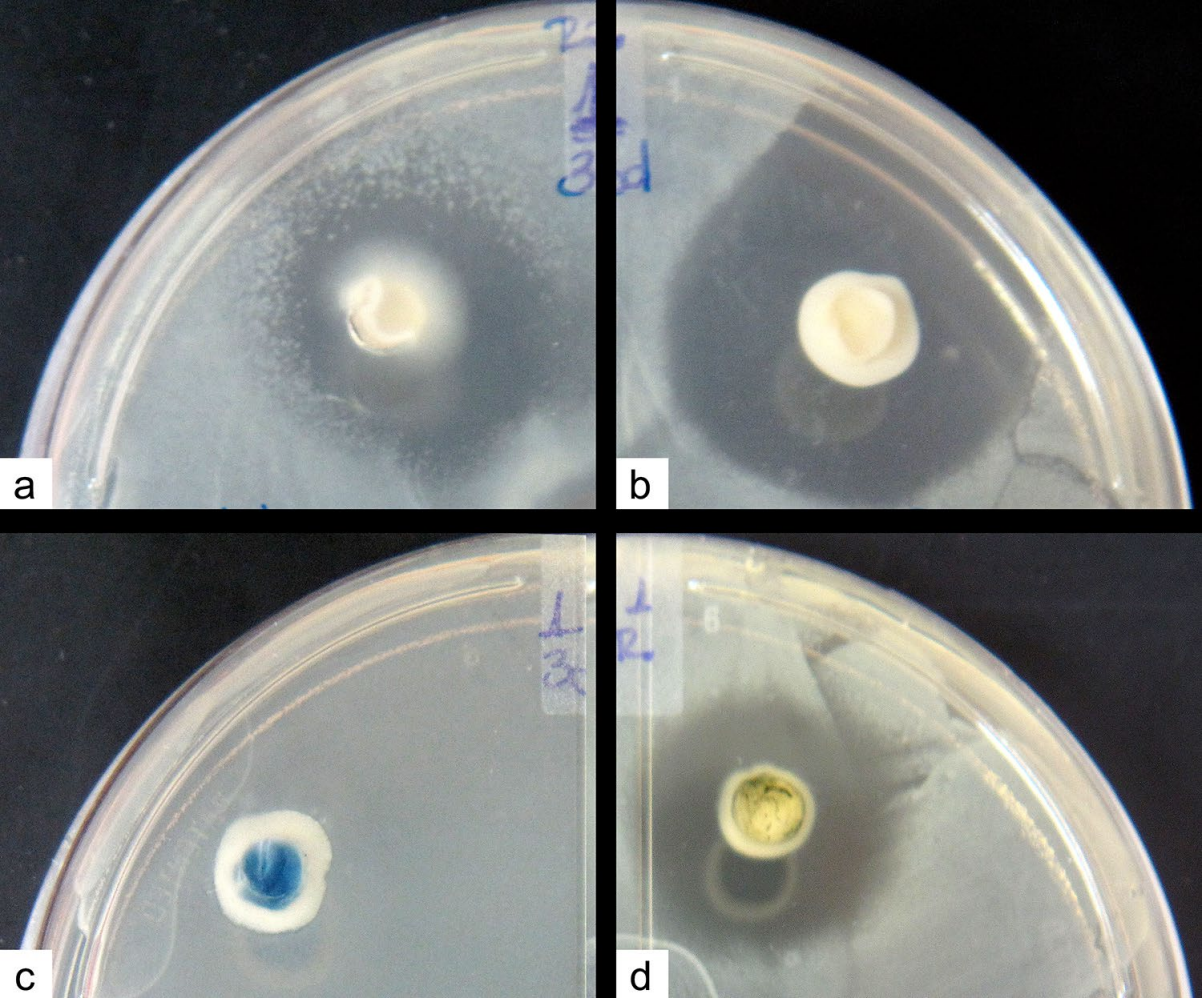
Bacterial growth Inhibition zone produced by fungi plugs exposed to *E. coli* during 30 days. (**a**) *Thelebolus* sp. (**b**) *Cryptococcus gilvescens*. (**c**) *Antarctomyces* sp. (**d**) *Penicillium* sp.

The antibacterial potential of the fungi was determined by the observation of bacterial growth inhibition zone around the mycelia plug (Fig. 4). Inhibition by *Thelebolus* sp. was only observed in the bioassay with *E. coli* at 15, 30 and 60 days of growth (Fig. 4a). At three different times of growth*, C. gilvescens* showed inhibitory effects to all Gram-positive and Gram-negative bacteria tested (Fig. 4b). *Antarctomyces* sp. did not show antibacterial activity against all the bacteria tested (Fig. 4c). Similar to *C. gilvescens*, *Penicillium* sp. showed inhibitory effects to all Gram-positive and Gram-negative bacteria tested at 15 and 30 days of growth (Fig. 4d).

In order to determine if growth time of the fungi (i.e*.* 15, 30 and 60 days) had a significant effect on the observed inhibition halo size, we conducted a Kruskal–Wallis and a Mann–Whitney statistical test to determine the difference between three and two different growth times, respectively.

Statistical analysis on the size of bacterial growth inhibition ring around mycelia plugs, previously incubated at 15, 30, and 60 days, before being exposed to the bacterial lawns, showed significant differences (p < 0.05) with *E. coli* and *Thelebolus* sp. plugs. On the other hand, *C. gilvescens* showed similar growth inhibition rings at all three growing periods for the four bacteria tested in the assay (p > 0.05). Similar results were obtained with bacteria exposed to 15 and 30 days grown of *Penicillium* sp. plugs (p > 0.05) (Supplementary file 3). *Antarctomyces* sp. was not included because it did not show antibacterial properties in our assays.

## Discussion

The kingdom Fungi is considered a key contributor to the biotechnology industry^42^, with several applications in textile, food, and pharmaceutics processes^43,44^. Valuable compounds with antitumor, antiparasitic and antibacterial activity have been identified in fungi from Antarctica^45–50^. Although they have great potential as novel source of compounds, the genetic diversity of microbes from this pristine and unique polar environment is largely unexplored^51^. In this study, we describe the genetic and morphological characterization of four soil fungi isolated from Fort William Point, Greenwich Island, near Pedro Vicente Maldonado Ecuadorian Antarctic Research Station, some of which showed bioactivity against relevant clinical bacterial isolates.

For the genetic characterization of our selected isolates, we used a phylogenetic tree based on the sequence of the ITS region, which is considered as the barcode for fungal taxonomy^52–54^. There is some disagreement whether this region alone has enough variability as a reliable species-specific identification marker^55–59^. This has been shown to be the case with some genera in the Ascomycota^60^. Some authors suggest that using the ITS region along with other protein-coding genes such as *RPB1* (RNA polymerase II largest subunit, regions E and F), *RPB2* (RNA polymerase II second largest subunit, regions 5–7), *Tsr1* (20S pre-rRNA processing protein), *Cct8* (subunit of the cytosolic chaperonin Cct ring complex) and MCM7 (Minichromosome Maintenance Complex Component 7) to identify fungal species of the same genera with low intraspecific variation^55^. ITS region together with other genes such as calmodulin and β-tubulin have been useful in deeper taxonomical studies to discriminate between the genera *Penicillium*^58^, which has proven difficult to classify among the fungi taxa^55^. Recently, the ITS combined with fragments of β-tubulin and *RPB2* were successfully used to identify a new species of *Antarctomyces*^61^*,* and to differentiate closely related fungi with low genetic variation^62^. However, other studies described β-tubulins as phylogenetically misleading, because they are present in the genome in multiple copies^63,64^. Species delimitation remains a challenging issue for closely related and cryptic fungal species^65,66^, and additional barcode markers, other than ITS, are being developed^59^.

In our study, we successfully confirmed the genus of our selected fungi with the use of a phylogenetic tree based on ITS sequencing. Isolates related to the Ascomycota group were confirmed as *Penicillium* sp., *Thelebolus* sp., and *Antarctomyces* sp. Identification to the species level for this group can be achieved with the implementation of additional gene sequence in upcoming studies. For a single isolate, the ITS sequencing allowed for species identification of the isolate T4-200-3B as *C. gilvescens*. Fungal morphological structures observed in this study were similar to previously descriptions for the same genera^67^. The integration of molecular data with other classification techniques such as morphology, ecology, new generation sequencing, and chemical profiling is nowadays our best set of tools to achieve a successful characterization of the fungi^61,68–71^.

Additionally, our phylogenetic analysis clearly separated the species of the Basidiomycota and Ascomycota phyla. *Antarctomyces* sp. and *Thelebolus* sp. segregated into sister clades that share an immediate common ancestor. These cryophilic genera have a slow generation time and thus accumulate only minor mutations, evolving slower than other species^72^. According to their geographic distribution, *Thelebolus* genus is known for its cryophilic nature and for its association with dung and guano^72^. Some species such as *T. globosus* and *T. ellipsoideus* are endemic to Antarctica, while others such as *T. microsporus* have a wider habitat, including Antarctica^16,72–74^. The genus *Antarctomyces* includes only two species, both native to the Antarctic continent^16,61,75^. Sharing the same phylum, *Penicillium* sp. clustered within the *P. lividum* and *P. odoratum* clade, and showed a strong bootstrap value with other species of the genus; all belonging to the section *Aspergilloides*^60,76^. The Basidiomycota phylum is represented by *Cryptococcus gilvescens*. This species distribution is restricted to cold environments, including the Antarctica^77,78^, where it is considered the most abundant genus of yeast^79^. *C. gilvescens* also showed a close relationship with *C. gastricus*, as previously reported^78^.

Bioactivity potential against pathogens is a promising application of the genetically diverse fungi of Antarctica. For instance, *C. gilvescens* and *Penicillium* sp. have shown antibiotic potential against Gram-negative bacteria, such as *E. coli* and *K. pneumoniae,* and Gram-positive bacteria, such as *E. faecalis* and *S. aureus*. This agrees in part with previous reports on the antibacterial activity of *Cryptococcus* species against Gram-positive bacteria^22,80^. In our study, *C. gilvescens* also showed antibacterial potential for Gram-negative bacteria. Additionally, *C. gilvescens* was reported to express extracellular lipolytic/esterasic activity, starch-degrading activity^81^, extracellular amylase, lipase, and protease activities^78^, anti-yeast activity^82^, and laccasse activity^83^. Various *Cryptococcus* isolated from Antarctic marine sediments had also exhibited lipase, esterase, and pectinase activity^84^.

In relation to species of *Penicillium* isolated from diverse polar ecosystems, such as marine sediments, deep-sea sediments, and sea-bed sediments, it is known that this genus has cytotoxic effects against cancer cell lines, anti-inflammatory effect, anti-allergic effect, antifungal and antibacterial activities^84^. A novel strain of *Penicillium* found in Antarctic soil showed production of three new indolyl diketopiperazine derivatives and seven known alkaloid compounds^85^. Some of these compounds had significant in vitro cytotoxic activity against cancer cell lines and one of them had antituberculosis activity^85^. An early study described nephrotoxicity in humans and strong antibiotic activity with *P. odoratum*^86^. This fungus produces the hazardous citrinin toxin, a mycotoxin that causes nephrotoxicity in humans^87–89^. Because the citrinin gene appears to be highly conserved within the genus *Penicillium*^90^, it is likely that citrinin is present in our *Penicillium* sp. isolate. *P. lividum* presented cytotoxic activity associated with the production of meroterpenoid compounds^91^. Furthermore, we report a *Penicillium* strain (*Penicillium* sp.) that produced antibacterial activity against Gram-negative and Gram-positive bacteria.

Previous studies have documented antitumoral^20^ and antibiotic potential in the *Thelebolus* genus, although the latter was less potent than *Penicillium*^35^. *Thelebolus* sp. from the Himalayas showed no antimicrobial activity against Gram-negative bacteria, but did exhibit antimicrobial activity against Gram-positive bacteria^92^. In contrast, *Thelebolus* sp. isolated in this study showed antibacterial activity against the Gram-negative bacteria *E. coli*. Several biotechnological applications have been attributed to *T. microsporus* due to the synthesis of linolenic acid, carotenoid pigments and extracellular *α*-amylase activity^93^. Lastly, our *Antarctomyces* isolate did not show any antibacterial activity against the tested bacteria in our in vitro assay conditions. Members of this genus, *A. psychrotrophicus* and *A. pellizariae* were attributed with potential biotechnological applications^16^. *A. psychrotrophicus* produced an antifreeze protein^94^, presented hydrocarbon biodegradation activity^95^ and showed antitumoral and antiprotozoal activity^96^. In addition, agar-block assays with *A. psychrotrophicus* described that this fungi has low antibacterial potential against *E. coli,* showing an inhibition growth zone between 7–10 mm^97^. On the other hand *A. pellizariae* produced a blue pigment with potential use in the food industry^61^.

To screen for bioactivity, this study used a low-cost in vitro assay adapted to the low temperature growth requirement of the fungi and the high temperature requirement for bacterial growth. This quick assay allowed us to detect bacterial growth inhibition zones around the fungi plugs as indicative of potential antibacterial activity. Without a complete knowledge of the environmental and nutrient requirements for the Antarctic fungi to produce bioactive compounds, we believe that this bioassay has its merit in detecting potential antibacterial metabolites that would have been missed otherwise. This bioassay may be extended to screen for antiviral and anticancer compounds, as well. Future studies will aim to isolate, identify, and characterize the putative bioactive compound(s). This work contributes to the preliminary description of soil fungi of Antarctica and to underscore its potential biotechnological applications and, thus, the importance of its environment conservation.

## Material and methods

### Soil sampling

**Figure 5:**
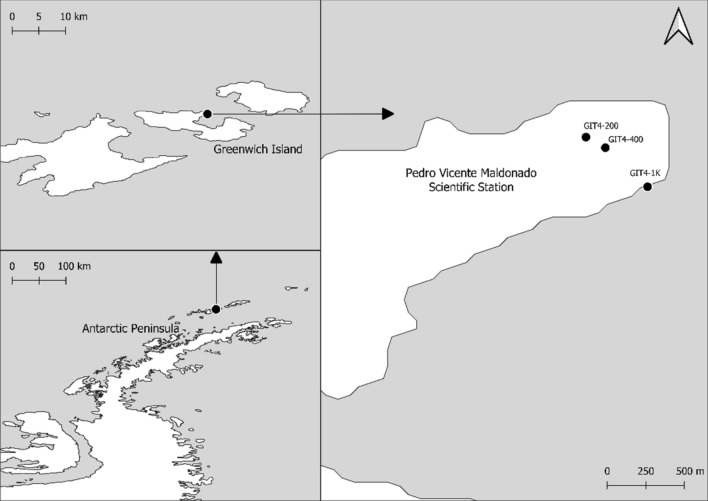
Location of Pedro Vicente Maldonado Scientific Station in Greenwich Island, Antarctica. The geographic location of the three land stations sampled are: GIT4-200 (62°26′53.9″S 59°44′07.7″W), GIT4-400 (62°26′58.7″S 59°43′58.9″W), and GIT4-1 K (62°27′16.4″S 59°43′39.8″W). The map was generated in QGIS version 3.10.14-A Coruña (https://www.qgis.org) using a geospatial vector of Antarctica's administrative boundaries obtained from http://www.diva-gis.org/.

The fungi evaluated in this study were isolated from soil samples collected in the Antarctic summer of 2008, near the “Pedro Vicente Maldonado” Antarctic Ecuadorian Scientific Station, located in Fort William Point, Greenwich Island. A total of three sites (stations GIT4-200, GIT4-400, and GIT4-1K) were sampled along a 1000 m linear transect (Fig. 5). At each sampling site, five soil sample replicates were collected with a sterile scoop in a 5 m radius from the registered GPS coordinate. The first 10 cm of soil surface from these five replicates were pooled and filtered with a 2 mm mesh. Soil samples were sealed in sterile polyethylene bags (Whirl-Pack) and transported in a cooler at 0–4 °C until their arrival to laboratory facilities at ESPOL (Guayaquil, Ecuador), where they were kept at 4 °C.

### Fungi isolation

The soil-plate method^98^ was used to grow the fungi on Potato Dextrose Agar, PDA (BD Difco™) with chloramphenicol (100 µg/ml) as the growth media. Under a vertical laminar flow cabinet, 0.1–0.3 g of soil were evenly dispersed on the solid media and incubated at 4 °C for a maximum of 12 days. Then, individual colonies were transferred to new PDA plates. The isolates analyzed in this report were codified as T4-200-3B, T4-400-5E, T4-1K-1G, and T4-1K-1A.

### Fungal DNA extraction

DNA was extracted from 20-day old fungi cultures according to a previously described protocol, with minor modifications^99^. Briefly, 200–500 mg of fungi mycelium was mixed with 200 mg of 0.1 mm Zirconia/Silica beads (Biospec) and 500 µl of a bead beating solution (0.1 M NaCl, 5% sodium dodecyl sulfate and 0.5 M Tris–HCl, pH 8) into a 1.5 ml microcentrifuge tube. The tube was vortexed at maximum speed for 10 min. The mixture was cleared by centrifugation at 11,000×*g* for 10 min. The supernatant was transferred to a new tube containing 200 mg of clean beads and vortexed. After centrifugation, the supernatant was transferred to a clean 1.5 ml microcentrifuge tube and mixed with an equal volume of phenol (Sigma-Aldrich), and chloroform-isoamyl alcohol (Sigma-Aldrich) (25:24:1). The mixture was briefly vortexed and centrifuged at 11,000×*g* for 5 min. The aqueous layer was transferred to a new tube and treated with an equal volume of chloroform: isoamyl alcohol (49:1) solution (Sigma-Aldrich), vortexed and centrifuged for 5 min at 10,000×*g*. The aqueous layer was transferred to a new tube and mixed with 2.5 volumes of isopropanol, incubated for 1 h at 4 °C, and centrifuged 14,000×*g* for 10 min. The DNA pellet was washed twice with ice-cold 70% ethanol, dried and resuspended in 0.1 × TE buffer (1 mM Tris–HCl, 0.1 mM EDTA pH 8).

### Amplification and cloning of the ITS region

The ITS region was amplified with Platinum Taq DNA polymerase (Invitrogen), using the universal primers ITS1 and ITS4^100^. PCR program consisted of 2 min at 94 °C, 35 cycles of 94 °C for 30 secs, 55 °C for 30 secs, 72 °C for 1 min, and a final extension of 3 min at 72 °C. Amplified products were resolved in a 1% agarose gel, stained with SYBR Safe (Invitrogen). The ITS fragment was cut from the gel, purified using High Pure PCR Product Purification Kit (Roche), cloned into a pGEM^®^-T Easy Vector (Promega), and sequenced by the Sanger method using SP6 and T7 universal primers (GENEWIZ, South Plainfield, NJ).

### Sequence analysis

The four ITS sequences from this study were matched with sequences from GenBank using the BLAST software (http://blast.ncbi.nlm.nih.gov/Blast.cgi). The final dataset included 43 sequences. They were then aligned using MAFFT^101^ with default settings. The final alignment was 737 bp long (Supplementary file 4). Sequences with complete information like species name, location and collection date were mainly selected for the phylogeny. The substitution model that best fit the data was selected using jModelTest 2.1.7^102,103^. The phylogenetic tree was constructed using the Maximum-likelihood method on MEGA Version 10.2.6^104^, using Kimura parameter-2 substitution model^105^ with uniform rate among sites, and 1000 Bootstrap replications.

### Microscopic observation

Microscopic structures were observed using a compound microscope with 1000 × magnification after 15 and 30 days of growth on PDA media. A small amount of fungal culture was removed from the edge of the colony using an inoculation loop and then stained with 60% of lactophenol blue solution on a microscopic glass slide. The software Motic Image Plus 2.0 was used to measure fungal structures.

### Antibiotic susceptibility tests

The clinical bacterial isolates used for the fungi antibacterial activity were previously diagnosed by classical antibiotic susceptibility tests using the Kirby Bauer method with the Agar Muller–Hinton media (Thermo Scientific™).

Because the Antarctic fungi were grown at low temperature and it is unknown the environmental conditions that may affect their potential antimicrobial activity, we performed the in vitro antibacterial assays on PDA plates at 4 °C and 37 °C. To this end, we first tested if an antibiotic resistant clinical isolate of *Klebsiella pneumoniae* was able to grow on PDA at 4 °C and 37 °C and still show antibiotic resistance. Bacteria streaked on PDA and Luria broth agar (LBA) were grown at 4 °C and 37 °C. The plates grown at 37 °C were incubated for 24 h, but the plates grown at 4 °C were incubated for 5 days, then the plates were transfer to 37 °C and incubated for further 24 h. On each plate antibiotic disks impregnated with 10 ug of imipenem and meropenem were deposited. Antibiotic resistance, depicted as clear rings around the antibiotic disks, were read after the 37 °C incubation in all treatments.

### Assay of antibacterial potential

The antibacterial potential for the fungi was determined using the mycelia plugs method^106^, with fungi isolates grown at 4 °C on PDA. The fungi were analyzed at three sampling times of growth i.e*. *15, 30, and 60 days. The clinical bacterial strains used in this assay were: *Escherichia coli, Klebsiella pneumoniae, Enterococcus faecalis* and *Staphylococcus aureus.* Plates with mycelia plugs and bacterial lawn were first incubated at 4 °C for 5 days on PDA to allow for fungi to grow and then transferred to 37 °C for 24 h for bacterial growth. The bioassays were performed with a minimum of three replicates, and the mean inhibition zone was calculated by measuring the border of the fungi colony to the border of the bacterial growth. This was photographed and measured in millimeters (mm) using the Motic Images Plus 2.0 software. The software SPSS 19 was used for the statistical analysis of the bacterial inhibition zone around the mycelia plug. The heterogeneity between days of growth was determined by applying Kruskal–Wallis and Mann–Whitney tests, with a statistical confidence level of 95%.

## Supplementary Information

Supplementary Information 1.

Supplementary Information 2.

Supplementary Information 3.

Supplementary Information 4.

## Data Availability

All the new sequences will be available from the GenBank (https://www.ncbi.nlm.nih.gov/genbank/) database with the accession codes: MZ958926, MZ958927, MZ958928, MZ958929. The datasets generated or analyzed in this study are included within the article and its supplementary files.
