# Supplementary material for: Antarctic fungi with antibiotic potential isolated from Fort William Point, Antarctica
Source: Sci Rep. 2022 Dec 12;12:21477. doi: 10.1038/s41598-022-25911-x (PMC9744802; doi:10.1038/s41598-022-25911-x)
Supplement: Supplementary file 1 — Supplementary Information 1. [file 41598_2022_25911_MOESM1_ESM.docx]

**Antarctic fungi with antibiotic potential isolated from Fort William Point, Antarctica**

Eunice Ordóñez-Enireb^1^, Roberto V. Cucalón^1,2^, Diana Cárdenas^1^, Nadia Ordóñez^1,3^, Santiago Coello^1^, Paola Elizalde^1,4^, Washington B. Cárdenas^1^*

^1^Laboratorio para Investigaciones Biomédicas, Facultad de Ciencias de la Vida, Escuela Superior Politécnica del Litoral, Guayaquil, Ecuador.

^2^Program in Ecology, Evolution, and Conservation Biology, University of Illinois at Urbana-
Champaign, Natural Resources Building 607 E. Peabody Dr., Champaign, IL 61820 USA.

^3^Biochemistry & Biosupport, Research & Development, Crop Science, Bayer AG, Monheim, Germany

^4^ Vaccine and Infectious Disease Organization (VIDO), University of Saskatchewan, 120 Veterinary Road, Saskatoon, Saskatchewan S7N5E3, Canada; School of Public Health, University of Saskatchewan, Saskatoon, Saskatchewan, S7N5E5, Canada

**Corresponding author: wbcarden@espol.edu.ec*

**Supplementary information file 1**

**Table S1.** Susceptibility test using the Kirby – Bauer Method of *Escherichia coli*. For each antibiotic tested, results show the halo diameter in millimeters (mm), and their specific interpretation as Susceptible (S), Intermediate (I), or Resistant (R).

| **Germ Id:** | *Escherichia coli* | | |  |
| --- | --- | --- | --- | --- |
| **Method:** | Kirby - Bauer (Disc Diffusion Test) | | |  |
| **Culture media:** | Agar Muller - Hinton | | |  |
|  |  | **Diameters (mm) breakpoints and interpretive categories** | | |
| **Antibiotics** | **Results** | **Susceptible** | **Intermediate** | **Resistant** |
| Ampicillin 10 ug | 6 mm (R) | $\geq$ 17 | 14 - 16 | $\leq$ 13 |
| Amoxicillin + Clavulanate 20/10 ug | 10 mm (R) | $\geq$ 18 | 14 - 17 | $\leq$ 13 |
| Ampicillin + Sulbactam 10/10 ug | 15 mm (S) | $\geq$ 15 | 12 - 14 | $\leq$ 11 |
| Piperacillin + Tazobactam  100/10 ug | 27 mm (S) | $\geq$ 21 | 18 - 20 | $\leq$ 17 |
| Cephalothin 30 ug | 16 mm (I) | $\geq$ 18 | 15 - 17 | $\leq$ 14 |
| Cefepime 30 ug | 32 mm (S) | $\geq$ 18 | 15 - 17 | $\leq$ 14 |
| Cefotaxime 30 ug | 32 mm (S) | $\geq$ 26 | 23 - 25 | $\leq$ 22 |
| Ceftriaxone 30 ug | 34 mm (S) | $\geq$ 23 | 20 - 22 | $\leq$ 19 |
| Cefoxitin 30 ug | 28 mm (S) | $\geq$ 18 | 15 - 17 | $\leq$ 14 |
| Ceftazidime 30 ug | 30 mm (S) | $\geq$ 21 | 18 - 20 | $\leq$ 17 |
| Cefuroxime 30 ug | 25 mm (S) | $\geq$ 23 | 15 - 22 | $\leq$ 14 |
| Aztreonam 30 ug | 34 mm (S) | $\geq$ 21 | 18 - 20 | $\leq$ 17 |
| Ertapenem 10 ug | 32 mm (S) | $\geq$ 23 | 20 - 22 | $\leq$ 19 |
| Imipenem 10 ug | 30 mm (S) | $\geq$ 23 | 20 - 22 | $\leq$ 19 |
| Meropenem 10 ug | 30 mm (S) | $\geq$ 23 | 20 - 22 | $\leq$ 19 |
| Gentamicin 10 ug | 20 mm (S) | $\geq$ 15 | 13 - 14 | $\leq$ 12 |
| Tobramycin 10 ug | 20 mm (S) | $\geq$ 15 | 13 - 14 | $\leq$ 12 |
| Amikacin 30 ug | 21 mm (S) | $\geq$ 17 | 15 - 16 | $\leq$ 14 |
| Minocycline 30 ug | 20 mm (S) | $\geq$ 16 | 13 - 15 | $\leq$ 12 |
| Ciprofloxacin 5 ug | 15 mm (R) | $\geq$ 21 | 16 - 20 | $\leq$ 15 |
| Levofloxacin 5 ug | 16 mm (I) | $\geq$ 17 | 14 - 16 | $\leq$ 13 |
| Norfloxacin 10 ug | 12 mm (R) | $\geq$ 17 | 13 - 16 | $\leq$ 12 |
| Nalidixic Acid 30 ug | 6 mm (R) | $\geq$ 19 | 14 - 18 | $\leq$ 13 |
| Sulfa-Trimethoprim 1.25/23.75 ug | 6 mm (R) | $\geq$ 16 | 11 - 15 | $\leq$ 10 |
| Fosfomycin 200 ug | 18 mm (S) | $\geq$ 16 | 13 - 15 | $\leq$ 12 |
| Nitrofurantoin 300 ug | 23 mm (S) | $\geq$ 17 | 15 - 16 | $\leq$ 14 |

*Note: BLEE non-producer bacterial strain

**Table S2.** Susceptibility test using the Kirby – Bauer Method of *Staphylococcus aureus*. For each antibiotic tested, results show the halo diameter in millimeters (mm), and their specific interpretation as Susceptible (S), Intermediate (I), or Resistant (R).

| **Germ Id:** | *Staphylococcus aureus* | | |  |
| --- | --- | --- | --- | --- |
| **Method:** | Kirby - Bauer (Disc Diffusion Test) | | |  |
| **Culture media:** | Agar Muller - Hinton | | |  |
|  |  | **Diameters (mm) breakpoints and interpretive categories** | | |
| **Antibiotics** | **Results** | **Susceptible** | **Intermediate** | **Resistant** |
| Oxacillin 1 ug | 22 mm (S) | $\geq$ 13 | 11 - 12 | $\leq$ 10 |
| Cefoxitin 30 ug | 28 mm (S) | $\geq$ 22 | - | $\leq$ 21 |
| Gentamicin 10 ug | 24 mm (S) | $\geq$ 15 | 13 - 14 | $\leq$ 12 |
| Amikacin 30 ug | 20 mm (S) | $\geq$ 17 | 15 - 16 | $\leq$ 14 |
| Azithromycin 15 ug | 23 mm (S) | $\geq$ 18 | 14 - 17 | $\leq$ 13 |
| Erythromycin 15 ug | 25 mm (S) | $\geq$ 23 | 14 - 22 | $\leq$ 13 |
| Tetracycline 30 ug | 25 mm (S) | $\geq$ 19 | 15 - 18 | $\leq$ 14 |
| Ciprofloxacin 5 ug | 30 mm (S) | $\geq$ 21 | 16 - 20 | $\leq$ 15 |
| Levofloxacin 5 ug | 27 mm (S) | $\geq$ 19 | 16 - 18 | $\leq$ 15 |
| Clindamycin 2 ug | 23 mm (S) | $\geq$ 21 | 15 - 20 | $\leq$ 14 |
| Sulfa-Trimethoprim 1.25/23.75 ug | 25 mm (S) | $\geq$ 16 | 11 - 15 | $\leq$ 10 |
| Chloramphenicol 30 ug | 21 mm (S) | $\geq$ 18 | 13 - 17 | $\leq$ 12 |
| Rifampin 5 ug | 20 mm (S) | $\geq$ 20 | 17 - 19 | $\leq$ 16 |
| Linezolid 30 ug | 30 mm (S) | $\geq$ 21 | - | $\leq$ 20 |

*Note: *Staphylococcus aureus,* oxacillin/methicillin susceptible

**Table S3.** Susceptibility test using the Kirby – Bauer Method of *Enterococcus faecalis*. For each antibiotic tested, results show the halo diameter in millimeters (mm), and their specific interpretation as Susceptible (S), Intermediate (I), or Resistant (R).

| **Germ Id:** | *Enterococcus faecalis* | | |  |
| --- | --- | --- | --- | --- |
| **Method:** | Kirby - Bauer (Disc Diffusion Test) | | |  |
| **Culture media:** | Agar Muller - Hinton | | |  |
|  |  | **Diameters (mm) breakpoints and interpretive categories** | | |
| **Antibiotics** | **Results** | **Susceptible** | **Intermediate** | **Resistant** |
| Ampicillin 10 ug | 17 mm (S) | $\geq$ 17 | 15 - 16 | $\leq$ 14 |
| Vancomycin 30 ug | 17 mm (S) | $\geq$ 17 | 15 - 16 | $\leq$ 14 |
| Tetracycline 30 ug | 6 mm (R) | $\geq$ 19 | 15 - 18 | $\leq$ 14 |
| Ciprofloxacin 5 ug | 25 mm (S) | $\geq$ 21 | 16 - 20 | $\leq$ 15 |
| Levofloxacin 5 ug | 26 mm (S) | $\geq$ 17 | 14 - 16 | $\leq$ 13 |
| Nitrofurantoin 300 ug | 24 mm (S) | $\geq$ 17 | 15 - 16 | $\leq$ 14 |
| Rifampin 5 ug | 13 mm (R) | $\geq$ 20 | 17 - 19 | $\leq$ 16 |
| Fosfomycin 200 ug | 25 mm (S) | $\geq$ 16 | 13 - 15 | $\leq$ 12 |
| Chloramphenicol 30 ug | 8 mm (R) | $\geq$ 18 | 13 - 17 | $\leq$ 12 |
| Linezolid 30 ug | 24 mm (S) | $\geq$ 23 | 21 - 22 | $\leq$ 20 |
| Gentamicin 120 ug | 6 mm (R) | $\geq$ 10 | 7 - 9 | 6 |
| Streptomycin 300 ug | 6 mm (R) | $\geq$ 10 | 7 - 9 | 6 |
| Imipenem 10 ug | 24 mm (S) | $\geq$ 12 | - | $\leq$ 12 |

**Table S4.** Susceptibility test using the Kirby – Bauer Method of *Klebsiella pneumoniae*. For each antibiotic tested, results show the halo diameter in millimeters (mm), and their specific interpretation as Susceptible (S), Intermediate (I), or Resistant (R).

| **Germ Id:** | *Klebsiella pneumoniae* | | |  |
| --- | --- | --- | --- | --- |
| **Method:** | Kirby - Bauer (Disc Diffusion Test) | | |  |
| **Culture media:** | Agar Muller - Hinton | | |  |
|  |  | **Diameters (mm) breakpoints and interpretive categories** | | |
| **Antibiotics** | **Results** | **Susceptible** | **Intermediate** | **Resistant** |
| Ampicillin 10 ug | 6 mm (R) | $\geq$ 17 | 14 - 16 | $\leq$ 13 |
| Amoxicillin + Clavulanate 20/10 ug | 6 mm (R) | $\geq$ 18 | 14 - 17 | $\leq$ 13 |
| Ampicillin + Sulbactam 10/10 ug | 6 mm (R) | $\geq$ 15 | 12 - 14 | $\leq$ 11 |
| Piperacillin + Tazobactam  100/10 ug | 15 mm (R) | $\geq$ 21 | 18 - 20 | $\leq$ 17 |
| Cephalothin 30 ug | 6 mm (R) | $\geq$ 18 | 15 - 17 | $\leq$ 14 |
| Cefepime 30 ug | 10 mm (R) | $\geq$ 18 | 15 - 17 | $\leq$ 14 |
| Cefotaxime 30 ug | 6 mm (R) | $\geq$ 26 | 23 - 25 | $\leq$ 22 |
| Ceftriaxone 30 ug | 6 mm (R) | $\geq$ 23 | 20 - 22 | $\leq$ 19 |
| Cefoxitin 30 ug | 20 mm (S) | $\geq$ 18 | 15 - 17 | $\leq$ 14 |
| Ceftazidime 30 ug | 10 mm (R) | $\geq$ 21 | 18 - 20 | $\leq$ 17 |
| Cefuroxime 30 ug | 6 mm (R) | $\geq$ 23 | 15 - 22 | $\leq$ 14 |
| Aztreonam 30 ug | 6 mm (R) | $\geq$ 21 | 18 - 20 | $\leq$ 17 |
| Imipenem 10 ug | 26 mm (S) | $\geq$ 23 | 20 - 22 | $\leq$ 19 |
| Meropenem 10 ug | 30 mm (S) | $\geq$ 23 | 20 - 22 | $\leq$ 19 |
| Gentamicin 10 ug | 20 mm (S) | $\geq$ 15 | 13 - 14 | $\leq$ 12 |
| Tobramycin 10 ug | 6 mm (R) | $\geq$ 15 | 13 - 14 | $\leq$ 12 |
| Amikacin 30 ug | 13 mm (R) | $\geq$ 17 | 15 - 16 | $\leq$ 14 |
| Minocycline 30 ug | 8 mm (R) | $\geq$ 16 | 13 - 15 | $\leq$ 12 |
| Ciprofloxacin 5 ug | 6 mm (R) | $\geq$ 21 | 16 - 20 | $\leq$ 15 |
| Levofloxacin 5 ug | 6 mm (R) | $\geq$ 17 | 14 - 16 | $\leq$ 13 |
| Norfloxacin 10 ug | 6 mm (R) | $\geq$ 17 | 13 - 16 | $\leq$ 12 |
| Nalidixic Acid 30 ug | 6 mm (R) | $\geq$ 19 | 14 - 18 | $\leq$ 13 |
| Sulfa-Trimethoprim 1.25/23.75 ug | 14 mm (I) | $\geq$ 16 | 11 - 15 | $\leq$ 10 |
| Fosfomycin 200 ug | 18 mm (S) | $\geq$ 16 | 13 - 15 | $\leq$ 12 |
| Nitrofurantoin 300 ug | 8 mm (R) | $\geq$ 17 | 15 - 16 | $\leq$ 14 |

*Note: BLEE producer bacterial strain
