# Supplementary material for: Antarctic fungi with antibiotic potential isolated from Fort William Point, Antarctica
Source: Sci Rep. 2022 Dec 12;12:21477. doi: 10.1038/s41598-022-25911-x (PMC9744802; doi:10.1038/s41598-022-25911-x)
Supplement: Supplementary file 2 — Supplementary Information 2. [file 41598_2022_25911_MOESM2_ESM.docx]

**Antarctic fungi with antibiotic potential isolated from Fort William Point, Antarctica**

Eunice Ordóñez-Enireb^1^, Roberto V. Cucalón^1,2^, Diana Cárdenas^1^, Nadia Ordóñez^1,3^, Santiago Coello^1^, Paola Elizalde^1,4^, Washington B. Cárdenas^1^*

^1^Laboratorio para Investigaciones Biomédicas, Facultad de Ciencias de la Vida, Escuela Superior Politécnica del Litoral, Guayaquil, Ecuador.

^2^Program in Ecology, Evolution, and Conservation Biology, University of Illinois at Urbana-
Champaign, Natural Resources Building 607 E. Peabody Dr., Champaign, IL 61820 USA.

^3^Biochemistry & Biosupport, Research & Development, Crop Science, Bayer AG, Monheim, Germany

^4^ Vaccine and Infectious Disease Organization (VIDO), University of Saskatchewan, 120 Veterinary Road, Saskatoon, Saskatchewan S7N5E3, Canada; School of Public Health, University of Saskatchewan, Saskatoon, Saskatchewan, S7N5E5, Canada

**Corresponding author: wbcarden@espol.edu.ec*

**Supplementary information file 2**

**Fig S1. Effect of temperature and PDA media on the susceptibility of *K. pneumoniae.***


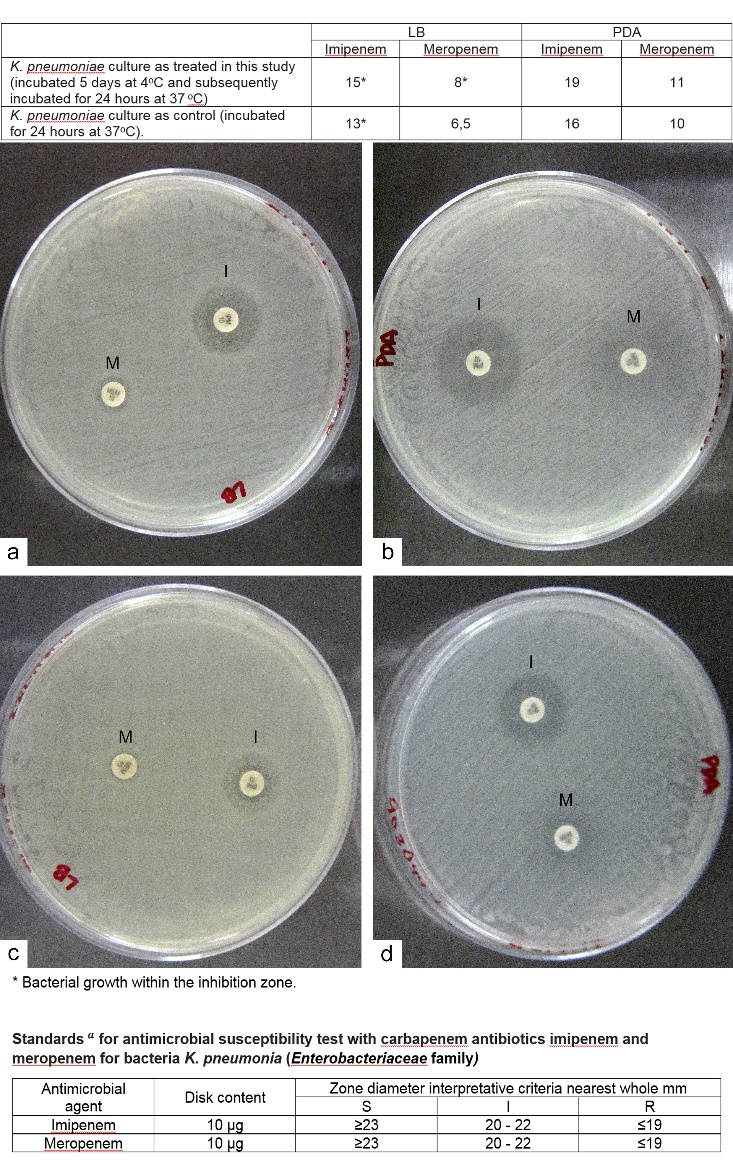
Results of the inhibition zone diameter (mm) formed by imipenem (I) and meropenem (M) in a *K. pneumoniae* culture in LB (bacteria media) and PDA (fungi media). Cultures were tested as treated in this study (incubated for 5 days at 4^o^C and subsequently incubated for 24 hours at 37 ^o^C) (a-b), and as control (incubated for 24 hours at 37^o^C) (c-d).

${}^{\boldsymbol{a}}$Clinical and Laboratory Standards Institute (CLSI). *Performance Standards for Antimicrobial Susceptibility Testing.* 28th ed. CLSI supplement M100 (ISBN 1-56238-838-X [Print]; ISBN 1-56238-839-8 [Electronic]). Clinical and Laboratory Standards Institute, 950 West Valley Road, Suite 2500, Wayne, Pennsylvania 19087 USA, 2018.
