# Supplementary material for: Antarctic fungi with antibiotic potential isolated from Fort William Point, Antarctica
Source: Sci Rep. 2022 Dec 12;12:21477. doi: 10.1038/s41598-022-25911-x (PMC9744802; doi:10.1038/s41598-022-25911-x)
Supplement: Supplementary file 3 — Supplementary Information 3. [file 41598_2022_25911_MOESM3_ESM.docx]

**Antarctic fungi with antibiotic potential isolated from Fort William Point, Antarctica**

Eunice Ordóñez-Enireb^1^, Roberto V. Cucalón^1,2^, Diana Cárdenas^1^, Nadia Ordóñez^1,3^, Santiago Coello^1^, Paola Elizalde^1,4^, Washington B. Cárdenas^1^*

^1^Laboratorio para Investigaciones Biomédicas, Facultad de Ciencias de la Vida, Escuela Superior Politécnica del Litoral, Guayaquil, Ecuador.

^2^Program in Ecology, Evolution, and Conservation Biology, University of Illinois at Urbana-
Champaign, Natural Resources Building 607 E. Peabody Dr., Champaign, IL 61820 USA.

^3^Biochemistry & Biosupport, Research & Development, Crop Science, Bayer AG, Monheim, Germany

^4^ Vaccine and Infectious Disease Organization (VIDO), University of Saskatchewan, 120 Veterinary Road, Saskatoon, Saskatchewan S7N5E3, Canada; School of Public Health, University of Saskatchewan, Saskatoon, Saskatchewan, S7N5E5, Canada

**Corresponding author: wbcarden@espol.edu.ec*

**Supplementary Information file 3**

**Table S5.** Descriptive statistics of the measurement of the inhibitory effects by the fungus species with antibacterial potential vs. *Escherichia coli.*

| **Antarctic microfungi vs. *Escherichia coli* inhibition halo diameter (mm)** | | | | | | | | | | |
| --- | --- | --- | --- | --- | --- | --- | --- | --- | --- | --- |
| Description | | *Cryptococcus gilvescens 200-3B* | | | *Penicillium* sp. *400-5E* | | | *Thelebolus* sp. *1K-1G* | | |
|  |  | 15 days | 30 days | 60 days | 15 days | 30 days | 60 days | 15 days | 30 days | 60 days |
| Media | | 9,52 | 8,98 | 10,36 | 7,21 | 6,05 | 0 | 7,71 | 5,62 | 1,92 |
| IC 95% Media | Inferior Limit | 8,21 | 6,24 | 7,74 | -5,21 | 3,82 | 0 | 3,25 | 3,79 | -0,4 |
|  | Superior Limit | 10,83 | 11,73 | 12,98 | 19,63 | 8,27 | 0 | 12,18 | 7,44 | 4,28 |
| Median | | 9,68 | 8,76 | 10,85 | 5,57 | 5,8 | 0 | 10,01 | 5,71 | 0,00 |
| Standard Deviation | | 0,82 | 1,73 | 2,11 | 5 | 0,89 | 0 | 3,59 | 1,73 | 2,82 |
| N | | 4 | 4 | 4 | 3 | 3 | 3 | 5 | 6 | 8 |
| Kruskal Wallis Test/ Mann Whitney Test Asymptotic Significance | | 0,495 | | | 0,827 | | | 0,018 | | |

**Table S6.** Descriptive statistics of the measurement of the inhibitory effects by the fungus species with antibacterial potential vs. *Klebsiella pneumoniae.*

| **Antarctic microfungi vs. *Klebsiella pneumoniae* inhibition halo diameter (mm)** | | | | | | | |
| --- | --- | --- | --- | --- | --- | --- | --- |
| Description | | *Cryptococcus gilvescens 200-3B* | | | *Penicillium* sp. *400-5E* | | |
|  |  | 15 days | 30 days | 60 days | 15 days | 30 days | 60 days |
| Media | | 8,457 | 10,27 | 8,383 | 4,34 | 4,288 | 0 |
| IC 95% Media | Inferior Limit | 6,643 | 7,538 | 5,478 | 1,374 | -1,443 | 0 |
|  | Superior Limit | 10,272 | 13,002 | 11,287 | 7,316 | 10,02 | 0 |
| Median | | 8,275 | 10,9 | 7,88 | 4,665 | 4,28 | 0 |
| Standard Deviation | | 1,141 | 1,1 | 2,339 | 2,831 | 2,307 | 0 |
| N | | 4 | 3 | 5 | 6 | 3 | 3 |
| Kruskal Wallis Test/ Mann Whitney Test  Asymptotic Significance | | 0,267 | | | 1 | | |

**Table S7.** Descriptive statistics of the measurement of the inhibitory effects by the fungus species with antibacterial potential vs. *Enterococcus faecalis.*

| **Antarctic microfungi vs. *Enterococcus faecalis* inhibition halo diameter (mm)** | | | | | | | |
| --- | --- | --- | --- | --- | --- | --- | --- |
| Description | | *Cryptococcus gilvescens 200-3B* | | | *Penicillium* sp. 400-5E | | |
|  |  | 15 days | 30 days | 60 days | 15 days | 30 days | 60 days |
| Media | | 7,06 | 6,20 | 4,19 | 9,53 | 7,09 | 0 |
| IC 95% Media | Inferior Limit | 4,44 | 5,94 | 0,56 | 6,26 | -1,96 | 0 |
|  | Superior Limit | 9,69 | 6,46 | 7,81 | 12,80 | 16,14 | 0 |
| Median | | 6,5 | 6,2 | 4,15 | 8,34 | 8,3 | 0 |
| Standard Deviation | | 1,65 | 0,10 | 2,28 | 3,12 | 3,64 | 0 |
| N | | 4 | 3 | 4 | 6 | 3 | 3 |
| Kruskal Wallis Test/ Mann Whitney Test  Asymptotic Significance | | 0,223 | | | 0,795 | | |

**Table S8.** Descriptive statistics of the measurement of the inhibitory effects by the fungus species with antibacterial potential vs. *Staphylococcus aureus.*

| **Antarctic microfungi vs. *Staphylococcus aureus* inhibition halo diameter (mm)** | | | | | | | |
| --- | --- | --- | --- | --- | --- | --- | --- |
| Description | | *Cryptococcus gilvescens 200-3B* | | | *Penicillium* sp. 400-5E | | |
|  |  | 15 days | 30 days | 60 days | 15 days | 30 days | 60 days |
| Media | | 12,04 | 11,13 | 10,15 | 10,61 | 10,16 | 0 |
| IC 95% Media | Inferior Limit | 10,44 | 6,95 | 8,18 | 3,12 | 4,36 | 0 |
|  | Superior Limit | 13,64 | 15,30 | 12,11 | 18,09 | 15,97 | 0 |
| Median | | 11,89 | 10,48 | 9,94 | 12,26 | 9,23 | 0 |
| Standard Deviation | | 1,00 | 2,62 | 1,58 | 3,01 | 2,34 | 0 |
| N | | 4 | 4 | 5 | 3 | 3 | 3 |
| Kruskal Wallis Test/ Mann Whitney Test  Asymptotic Significance | | 0,355 | | | 0,827 | | |
